# Mobile Mammography Participation Among Medically Underserved Women: A Systematic Review

**DOI:** 10.5888/pcd15.180291

**Published:** 2018-11-15

**Authors:** Suzanne Vang, Laurie R. Margolies, Lina Jandorf

**Affiliations:** 1 Department of Population Health Science and Policy, Icahn School of Medicine at Mount Sinai, New York, New York; 2 Department of Radiology, Icahn School of Medicine at Mount Sinai and Mount Sinai Hospital, New York, New York

## Abstract

**Introduction:**

Although breast cancer deaths have declined, the mortality rate among women from medically underserved communities is disproportionally high. Screening mammography is the most effective tool for detecting breast cancer in its early stages, yet many women from medically underserved communities do not have adequate access to screening mammograms. Mobile mammography may be able to bridge this gap by providing screening mammograms at no cost or low cost and delivering services to women in their own neighborhoods, thus eliminating cost and transportation barriers. The objective of this systematic review was to describe the scope and impact of mobile mammography programs in promoting mammographic screening participation among medically underserved women.

**Methods:**

We searched electronic databases for English-language articles published in the United States from January 2010 through March 2018 by using the terms “mobile health unit,” “mammogram,” “mammography,” and “breast cancer screening.” Of the 93 articles initially identified, we screened 55; 16 were eligible to be assessed and 10 qualified for full text review and data extraction. Each study was coded for study purpose, research design, data collection, population targeted, location, sample size, outcomes, predictors, analytical methods, and findings.

**Results:**

Of the 10 studies that qualified for review, 4 compared mobile mammography users with users of fixed units, and the other 6 characterized mobile mammography users only. All the mobile mammography units included reached underserved women. Most of the women screened in mobile units were African American or Latina, low income, and/or uninsured. Mobile mammography users reported low adherence to 1-year (12%–34%) and 2-year (40%–48%) screening guidelines. Some difficulties faced by mobile clinics were patient retention, patient follow-up of abnormal or inconclusive findings, and women inaccurately perceiving their breast cancer risk.

**Conclusion:**

Mobile mammography clinics may be effective at reaching medically underserved women. Adding patient navigation to mobile mammography programs may promote attendance at mobile sites and increase follow-up adherence. Efforts to promote mammographic screening should target women from racial/ethnic minority groups, women from low-income households, and uninsured women. Future research is needed to understand how to best improve visits to mobile mammography clinics.

## Introduction

With the exception of skin cancers, breast cancer is the most commonly diagnosed cancer in US women, accounting for 15.3% of new cancers (1). Although breast cancer deaths are declining, the mortality rate among women from medically underserved (hereafter, “underserved”) communities is disproportionally high compared with rates in the general US female population. For example, breast cancer death rates among African American women (28.7 per 100,000) are 37% higher than the national average (20.9 per 100,000), and women living in poverty are 1.46 times more likely to die from breast cancer than those who are more affluent (1,2). Underserved women are defined as women with poor access to health care; compared with women without barriers to health care access, they are disproportionately from racial/ethnic minority groups, have a relatively low income, and have less education (3). Low-income women from racial/ethnic minority groups are 1.5 times as likely as their non-Hispanic white counterparts and 1.3 as likely as their higher-income counterparts to be diagnosed with late-stage cancers, which in part explains their lower breast cancer survival compared with the general US female population (4,5). Screening mammography is the only proven tool for detecting breast cancer in its early stages, yet many women from underserved communities do not adequately use mammograms (5,6). For example, African American mammographic screening rates are 19% lower than rates for non-Hispanic white women, and women living in high-poverty areas are 50% less likely than women living in higher-income areas to have received a mammogram in the previous 2 years (7,8). Multiple factors contribute to the lower screening rates of underserved women, ranging from socioeconomic and cultural factors to health system barriers (9,10).

Mobile mammography is one strategy for improving access to screening mammography. These programs typically provide screening mammograms at no cost or low cost and deliver services to women in their neighborhoods, eliminating cost and transportation barriers. Although mobile mammography has been used for more than 3 decades, little is known about participation among underserved women. The purpose of this systematic review was to describe the scope and impact of mobile mammography programs in promoting mammographic screening participation among underserved women.

## Methods

### Data sources

We conducted an electronic search of PubMed, MEDLINE, CINAHL, Embase, and PsycINFO to identify journal articles published in the English language, in the United States, from January 1, 2010, through March 31, 2018, that reported on observational or intervention studies promoting screening mammography using a mobile mammography clinic. Search terms used were combinations of Medical Subject Heading (MeSH) and keyword terms: “mobile health unit,” “mammogram,” “mammography,” and “breast cancer screening.” We also conducted a title search on Google Scholar using the following terms: “mobile mammogram,” “mobile mammography,” “mammogram van,” “mammography van,” “mammogram bus,” “mammography bus,” and “mobile breast screening.” We adhered to the standards for systematic reviews as outlined in the PRISMA statement (11).

### Study selection

Our interest in conducting this review was to understand the impact of mobile mammography on screening participation among underserved women in the United States. We considered articles eligible for initial inclusion if they focused on mobile mammography and provided a scientific abstract. Because the United States has a unique health care system that may influence the uptake of mammography among underserved women, we limited articles to studies conducted in the United States. We also restricted our selection to studies that included any group of underserved women in their sample. Because most mobile mammography programs are targeted to underserved women, we did not conduct a keyword search for underserved women but instead manually examined the abstracts to ascertain whether this criterion was met. Any of the following were considered underserved: any racial/ethnic minority group (eg, African American/black, Latina/Hispanic, Asian/Pacific Islander, Native American/American Indian) and regardless of race/ethnicity, any low-income, uninsured or underinsured, disadvantaged, rural, urban, or disabled group. We included an article if the research design was an observational study, clinical trial, or secondary data analysis. We excluded case reports, review articles, and editorials. We also excluded articles discussing only logistics of developing and/or maintaining mobile mammography programs (eg, cost-effectiveness, van development, staff training).

Two authors (S.V. and L.J.) performed independent reviews of the identified titles and abstracts to assess how well they fulfilled the inclusion criteria for full-text review. All disagreements were resolved by consensus. Next, these authors reviewed full-text articles and confirmed with another author (L.R.M.) which articles to include for full data extraction. One author (S.V.) reviewed bibliographies for all articles during full-text review to identify additional relevant articles. 

### Data extraction

Two reviewers (S.V. and L.J.) independently extracted data. The included studies were first categorized by whether the study 1) compared outcomes between mobile and fixed sites or 2) focused only on outcomes from mobile mammography sites. For each study, the 2 reviewers initially coded the study’s location, population targeted, sample size, research design, purpose, major findings, data collection, screening outcomes, predictors, and analytical methods. The authors also extracted data on screening guidelines, recency of screening, and adherence rates. Data were tabulated in the following categories: study location, underserved group targeted, sample size, research design, screening guidelines and recency of screening, adherence rate, study purpose, and major findings. Furthermore, in summarizing the results, the authors synthesized the literature and reported on findings according to the following topics: mobile versus fixed sites, sociodemographic characteristics of mobile unit users, repeat visits, screening adherence and recency of screening, screening outcomes, and perceived risk. All categories were approved by all authors. One author (L.R.M.) reviewed and confirmed extracted data. Because our systematic review was descriptive and not limited to clinical trials, we did not assess risk of bias or study quality.

## Results

Searches returned 93 articles published from January 1, 2010, through March 31, 2018; we removed 38 duplicates, and 55 articles were screened (Figure). After eliminating 39 for not meeting study criteria, 16 were fully assessed for eligibility. Of these, 5 were excluded because they did not examine women’s participation in mobile mammography, and 1 was removed because it was a dissertation under embargo. Of the 93 articles identified initially, 10 articles (11%) met selection criteria for full-text data extraction (Table). Four studies compared outcomes between mobile sites and fixed sites, whereas 6 studies focused on characteristics or outcomes from mobile sites only. One study took place in California, 3 in the Midwest, and 6 in the South. Three studies drew participants from urban areas, 4 from rural areas, and 3 from both urban and rural areas. The majority of underserved women targeted were African American (n = 6 studies) or Hispanic (n = 5 studies). Three studies targeted underserved women from Appalachia. One study focused on American Indians. The most common study designs were retrospective chart reviews (n = 4) and cross-sectional surveys (n = 4). Sample sizes ranged from 11 (women in qualitative focus groups) to more than 21,000 (a review of electronic medical records). All studies described the characteristics of women attending mobile mammography clinics.

**Figure:**
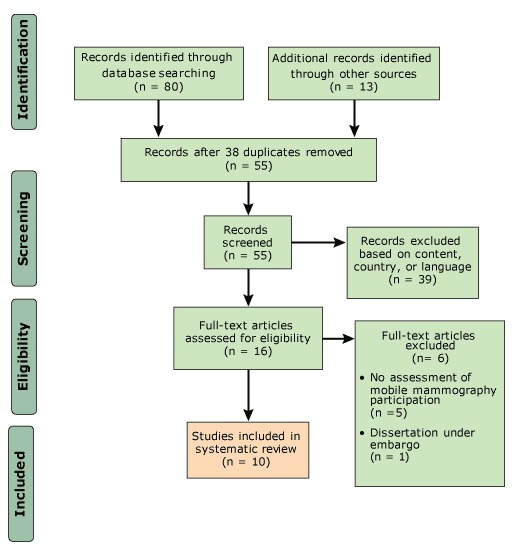
The process of including and excluding articles analyzed in a systematic review of mobile mammography among medically underserved women, United States, January 2010–March 2018.

**Table:** Results of Studies Examining Medically Underserved Women’s Participation in Mobile Mammography, United States, January 2010–March 2018

Study Location	Underserved Group Targeted and Sample Size	Research Design	Screening Guideline and Recency of Screening	Adherence Rate	Study Purpose	Major Findings
**Studies That Compared Mobile Sites With Fixed Sites**						
Chen et al, 2016 (12)						
Santa Clara County, California: mobile mammography operated by nonprofit community health centers; fixed unit operated by county hospital	Uninsured or underinsured Latina, Asian, or African American women (n = 11). Non-Hispanic white women not included.	Mixed methods: focus groups and a demographic survey	Not reported	Not reported	Examine women’s perceptions of mobile mammography and fixed mammography	Women’s perceptions of mobile mammography: • Concerned about quality of images • Felt technologists were less meticulous at mobile sites than at fixed sites • Experienced better communication at mobile site (eg, notification of long wait times, telephone call reminders)
Fayanju et al, 2014 (13)						
St. Louis and southeastern Missouri: mobile mammography operated by university hospital; fixed unit operated by academic medical center	Low-income African American and Hispanic women; non-Hispanic white women also included (n = 9,082).	Cross-sectional survey: 6-item questionnaire about women’s mammography experience	Not reported	Not reported	Investigate perceived barriers to use of screening mammograms	Three most commonly perceived barriers to screening mammography were • Fear of cost • Fear of mammogram-associated pain • Fear of getting bad news
						Fear of mammogram-related pain was more likely to be reported among • Women screened on van (OR, 1.63) than among women at fixed clinic sites • Black (OR, 1.32) and Hispanic (OR, 1.05) women than among non-Hispanic white women; and • Uninsured women than among insured women (OR, 1.39)
						Black (OR, 2.46) and Hispanic (OR, 2.98) women were also more likely to report fear of receiving bad news than were non-Hispanic white women.
Stanley et al, 2017 (14)						
Charleston, South Carolina, and surrounding areas: mobile mammography unit operated by university hospital; fixed unit operated by academic medical center	Hispanic and African American women; non-Hispanic white and “other” racial/ethnic women also included (n = 1,433 at mobile site; n = 1,434 at fixed site).	Retrospective review of electronic medical records	Past 1 year	Mobile, 34.5%; fixed, 56.9%	Evaluate characteristics of women who use mobile vs fixed mammography	• Mobile site had a higher recall rate than fixed site (16% vs 13%) • Among patients with a BI-RADS category 0, mobile unit patients were more likely than fixed-clinic users to not adhere to follow-up (17.0% at mobile unit vs 2.6% at fixed site)
Vyas et al, 2013 (15)						
West Virginia: mobile mammography unit operated by university hospital; fixed unit operated by university medical center	Low-income and/or uninsured Appalachian women; Appalachian women from other income groups and insured Appalachian women also included (n = 1,161 at mobile unit; n = 1,104 at fixed unit).	Cross-sectional survey: questionnaire consisting of personal health history, menstrual and pregnancy history, family history of cancer, cancer risk assessment and screening history, views on breast cancer screening, breast cancer awareness, preventive care and wellness history, nutrition and exercise history, dental, smoking and alcohol consumption history, and demographics	Past 2 years	Mobile, 48.2%; fixed, 92.3%	Compare characteristics of women who use mobile unit vs fixed mammography	Women using mobile unit, compared with women using the fixed unit, were • More likely to be obese (OR, 1.87), smoke (OR, 1.77), or not visit a doctor in the past year (OR, 1.38) • Less likely to report consuming alcohol (OR, 0.54) or having transportation barriers (OR, 0.50) • More likely to have lower adherence to other preventive screenings (OR, 1.60) and to have lower levels of perceived 5-year risk of developing breast cancer (OR, 0.48)
**Studies Examining Mobile Mammography Sites Only**						
Brooks et al, 2013 (16)						
Jefferson County, Kentucky: mobile mammography unit operated by private hospital	Uninsured African American and Hispanic women; non-Hispanic white women also included (n = 3,923).	Retrospective review of electronic medical records	Past 5 years	29%	Evaluate mammographic screening outcomes and their predictors	Women with abnormal mammograms (BI-RADS category 4,5, or 6) were more likely than women with normal mammograms (BI-RADS category 1, 2, or 3) to be • Aged <50 y (OR, 1.65) • Hispanic (OR, 1.87) • Uninsured (OR, 1.63) And less likely to report • Not smoking (OR, 0.65) • Not having a relative diagnosed with cancer before age 50 (OR, 0.64).
						Women with BI-RADS category 0 mammograms were less likely than women with BI-RADS category 1, 2, or 3 to • Have been screened within the past 5 years (OR, 0.64) • Be African American (OR, 0.68) And were more likely to not have a primary care physician (OR, 1.50)
Drake et al, 2015 (17)						
St. Louis, Missouri: mobile mammography unit operated by university hospital	African American women; non-Hispanic white women also included (n = 8,450).	Secondary data analysis: mammography outreach registry with data on patient demographics and quality of mammography experience	Not reported	Not reported	Identify factors associated with repeat use of mobile mammography	Repeat visits were more likely to occur among women who were • Aged 50–65 (OR, 1.15) vs aged 40–50 • Uninsured (OR, 1.32) vs insured • African American (OR, 1.26) vs non-Hispanic white Repeat visits were less likely among women who were • Aged <40 (OR, 0.34) vs aged 40–50 • Unemployed (OR, 0.86) vs employed • Living in a rural area (OR, 0.49) vs suburban
LeMasters et al, 2014 (18)						
West Virginia: mobile mammography operated by university hospital	Low-income or uninsured Appalachian women; Appalachian women from other income groups and insured Appalachian women also included (n = 1,182).	Cross-sectional survey: questionnaire on demographics, personal health history, menstrual and reproductive history, family history of cancer, breast cancer risk perceptions, breast cancer knowledge, perceived benefits and barriers to mammography, anxiety about developing breast cancer, clinical preventive care, health status, and health behavior/lifestyle	Past 1 year and 2 years	Past 1 year: 11.8%; past 2 years: 48.0%	Describe characteristics of women who responded “don’t know” when asked about their perceived 5-year risk of developing breast cancer	Women who responded “don't know” to their perceived 5-year breast cancer risk, compared with women who made an accurate or inaccurate response, • Were from lower-income families • Had less education • Were uninsured or had Medicare • Reported less knowledge about breast cancer
Mizuguchi et al, 2015 (19)						
Jefferson County, Kentucky, and surrounding areas: mobile mammography operated by university hospital	Uninsured African American or Hispanic women; non-Hispanic white women and “other” racial/ethnic group also included (n = 21,857).	Retrospective chart review: electronic medical records and data from patient information history form	Not reported	Not reported	Assess repeat use of mobile mammography	• Most (54%) patients used mobile mammography only once. • African American and Hispanic women used mobile mammography at a disproportionately higher rate than non-Hispanic white women. • Uninsured women made up the largest percentage (43.1%) of mobile mammography users. • African American women (30.5%) and women with Medicare insurance (31.5%) had the highest frequency of ≥3 repeat screenings at the mobile clinic among all racial/ethnic groups studied (non-Hispanic white, Hispanic, other) and other insurance types (private, Medicaid, uninsured), respectively. • Hispanic women were least likely group to be repeat users
Roen et al, 2013 (20)						
Reservations in North Dakota, South Dakota, Nebraska, and Iowa: mobile mammography operated by Indian Health Service	American Indian women only (n = 1,771).	Retrospective chart review of mammogram records	Past 2 years	40%	Determine adherence to screening mammography	• Women aged 41–49 were less likely (OR, 0.65) to have been adherent to screening mammogram guidelines compared with women aged 65 or older. • American Indian women using mobile mammography reported lower adherence (39.9%) than did American Indian women (59.8%), non-Hispanic white women (77.6%), and all ethnicities combined (74.3%) in the Breast Cancer Surveillance Consortium.
Vyas et al, 2012 (21)						
West Virginia: mobile mammography operated by university hospital	Low-income, uninsured Appalachian women; Appalachian women from other income groups and insured Appalachian women also included (n = 686).	Cross-sectional survey: questionnaire on personal health history, menstrual and pregnancy history, family history of cancer, cancer risk assessment and screening history, views on breast cancer screening, breast cancer awareness, preventive care and wellness history, nutrition and exercise history, dental, smoking and alcohol consumption history, and demographics	Past 2 years	46%	Identify predictors of adherence in women who use mobile mammography	Women who were adherent were more likely to • Be older (OR, 3.88) • Be extremely or morbidly obese (OR, 1.93 and 2.36, respectively) • Have a family history of breast cancer (OR, 1.87) • Have a history of breast problems (OR, 1.90) • Have low knowledge of screening (OR, 2.17) And less likely to: • Be nonadherent to Papanicolaou (Pap) guidelines (OR, 0.16) • Have low rates of completion of other preventive screenings (OR, 0.52)

Abbreviations: BI-RADS, Breast Imaging Reporting and Data System; OR, odds ratio.

**Mobile sites versus fixed sites.** Of the 4 studies that compared mobile sites with fixed sites, 2 studies (14,15) examined the rate of adherence to screening guidelines and found that mobile mammography users had lower previous rates of adherence than users at fixed sites (Table). Using a 1-year guideline for mammographic screening, one study (14) reported that 34% of mobile mammography users were currently adherent to screening guidelines, whereas 57% of users at fixed sites were. Similarly, another study (15) found that women using mobile mammography were 91% less likely than users at fixed sites to have had a screening mammogram within the past 2 years. Sociodemographically, mobile mammography users were more likely than users at fixed sites to be uninsured, have incomes below $25,000, be African American or Latina, and report being single (14,15). Furthermore, mobile mammography users were more likely to be obese and smoke and less likely to be adherent to other screening guidelines (eg, Papanicolaou [Pap] test) or have seen a primary care provider in the past year.

When we examined differences between how mobile users and fixed-site users evaluated mammography services, we found that mobile mammography users reported better communication from mobile-site staff members (eg, providing reasons for delays, appointment reminders) than users reported at fixed sites.

One study (13) identified 3 commonly cited barriers to screening mammography among users at mobile and fixed sites: fear of cost, fear of mammogram-associated pain, and fear of bad news. Mobile mammography users were less likely (odds ratio [OR], 0.77) than fixed-site users to report fear of receiving bad news, but they were more likely (OR, 1.63) to report fear of mammogram-related pain. Black women and Hispanic women, regardless of screening site, were more likely than non-Hispanic white women to report fear of mammogram-related pain (OR, 1.32 and 1.05, respectively) and fear of receiving bad news (OR, 2.46 and 2.98, respectively). Uninsured women from both types of sites were more likely (OR, 1.39) than insured women to report fear of mammogram-related pain.

**Sociodemographic characteristics of mobile unit users. **All 10 studies indicated that women who used mobile mammography clinics were from underserved groups. Most studies reported that users of mobile mammography identified as African American (48%–62%) or Hispanic (4%–11%) (13,14,16,17,19), had incomes below $25,000 (13,15,18,21), and/or were uninsured (14–16,18–20). One study documented use of mobile mammography by American Indians residing in the Northern Plains (20). Three studies included both urban and rural areas. One study (14) that compared mobile clinics with fixed sites found that although mobile mammography reached a greater percentage of urban women than rural women (71% vs 29%), a significantly greater proportion of urban women attended the stationary clinic than the mobile clinic (80% urban women at fixed sites vs 71% urban women at the mobile clinic, *P *< .001). Another study (17) reported women from rural areas were less likely (OR, 0.34) than women in suburban areas to make repeat use of mobile mammography. A third study (13) did not assess urban–rural differences in participation.

**Repeat visits.** Two studies reported on characteristics of women who made repeat use of mobile mammography (17,19). Both studies documented that most mobile mammography users did not return for future screenings: 54% to 75% of users in these studies used the mobile unit only once during an 8- to 10-year period. Repeat visits were more likely among African American women than among women of another race/ethnicity (non-Hispanic white, Hispanic, or other), among women who were uninsured or had Medicare than among women who had private insurance or Medicaid, or among women aged 50 to 65 than among women in other age groups group (<40, 40–50, >65). Hispanic women were the least likely racial/ethnic group to make repeat visits to mobile mammography units (17,19).

**Screening adherence and recency of screening**. Overall, rates of screening adherence among users of mobile mammography were low. Two studies found that only 12% to 34% of mobile mammography users had had a screening mammogram in the past year (14,18). Four studies found that 40% to 48% of mobile mammography users adhered to 2-year screening guidelines (15,18,20,21). Another study documented that only 29% of mobile mammography users had completed a screening mammogram within the past 5 years (16).

Adherence to screening was less common among women aged 40 to 49 than among women in other age groups (20,21). Women were also less likely to adhere to screening guidelines if they were also noncompliant with Pap testing guidelines or other preventive screenings (21). Higher adherence rates were found among women who were extremely or morbidly obese, had a family history of breast cancer, had previous breast problems, or had less knowledge of breast health (21).

**Screening outcomes.** Two studies documented screening outcomes among mobile mammography users (14,16). Mobile mammography users had higher rates of being recalled for further imaging than users at fixed sites (16% vs 13%). Compared with users at fixed clinics, mobile mammography users were more likely to have a mammogram categorized as 0 in the Breast Imaging Reporting and Data System (BI-RADS) (which means that additional imaging evaluation and/or comparison to a previous mammograms is needed), particularly women whose last screening was 5 years ago or more, women without a primary care provider, and women who identified as Hispanic or white (14,16). Additionally, women who needed follow-up were more likely to be Hispanic than non-Hispanic, younger than 50, have no insurance, smoke, or have a family member who received a cancer diagnosis when aged 50 or younger (16). Among women whose mammogram was categorized as BI-RADS 0, mobile mammography users were less likely than users of fixed sites to return for additional imaging: 17% of mobile site users and 3% of fixed site users did not return.

**Perceived risk.** Two studies reported on mobile mammography users’ perceived 5-year risk of developing cancer (15,18). They were more likely than fixed site users to perceive a lower 5-year risk of developing breast cancer (15). One-third of mobile mammography users reported “don’t know” when asked to assess their perceived risk (18). Women who responded they did not know their perceived risk were more likely to have lower incomes, be less educated, have Medicare or be uninsured, and to have less knowledge about breast cancer than women who reported either less perceived risk or greater perceived risk. Mobile mammography users who accurately reported their risk, compared with women who inaccurately reported their risk, tended to report lower perceived risk, were more educated, never had a biopsy, did not have a family history of cancer, were younger at first childbirth, and/or not nulliparous (18).

## Discussion

Findings from the 10 studies examined in this review suggest that mobile mammography programs do reach underserved women. Most women using mobile mammography lacked insurance and were from racial/ethnic minority backgrounds (mainly African Americans and Hispanic) and low-income households. Mobile mammography users also tended to have low rates of adherence to screening guidelines: 12% to 34% adhered to the 1-year guideline and 40% to 48% adhered to the 2-year guideline. These rates are lower than national rates of 50% for the 1-year guideline and 64% for the 2-year guideline and well below the Healthy People 2020 goal of having 81% of women aged 50 to 74 screened (22,23). Our findings highlight disparities in breast cancer screening among underserved women and underscore the importance of using outreach strategies such as mobile mammography to improve access and adherence to screening mammography guidelines.

Women aged 40 to 49 are less likely than women in older age groups to adhere to screening guidelines (24). Controversy surrounding the age at which mammograms should begin may influence the screening practices of younger women (25). Some evidence suggests that underserved women, particularly African American women, would benefit from starting screening at age 40 or even earlier, contrary to recommendations of the US Preventive Services Task Force (26). Thus, it is critical to increase breast cancer risk knowledge among underserved women to resolve breast cancer disparities.

Our review revealed that mobile mammography users typically did not return to the same mobile unit for additional screenings and that Hispanic women were the least likely racial/ethnic group to make a repeat visit. Mobile mammography users may have a more transient lifestyle than users of fixed sites; many are from low-income households, and low income can result in transient living situations. Furthermore, some Hispanic women, such as those employed in the cattle and harvesting industries, may be more likely to move around to find work and thus be less likely to return for repeat screenings (23,27). Concerns about image quality and poor service could also discourage women from revisiting mobile clinics (12). Further examination of women’s perceptions of mobile mammography showed that although some users initially had negative views about the quality of mobile mammography services, users often reported more positive experiences than women at fixed clinics (12). Providing patient navigation and appointment reminders may help promote attendance at mobile mammography clinics (12). Thus, efforts should be made to educate communities about the quality of mobile mammography services to improve participation and retention. More research is needed to explore factors associated with nonrepeat visits among women using mobile mammography.

The studies we examined reported that mobile mammography users were more likely than fixed-clinic users to be recalled for additional imaging, particularly women who had not been screened in 5 years or more, women without a primary care provider, and women who identified as Hispanic or white. Higher recall rates among these women may have been due to a lack of previous images, making it difficult to determine whether findings were stable or required further evaluation. No study identified the type of mammographic technology used (eg, film-screen, full-field digital, digital breast tomosynthesis); thus, it is uncertain whether differences in mammographic technology could explain differences in recall rates among sites. Mobile mammography users who required additional imaging were also less likely than their fixed-site counterparts to adhere to follow-up. Health education, text reminders, and patient navigation are promising strategies for improving compliance and should be explored by mobile mammography programs (12,28). The lack of diagnostic imaging on mobile units may pose access barriers (eg, transportation, cost) to underserved women needing follow-up examinations. Adding diagnostic mammography capability and ultrasound units on mobile units could expand reach to underserved women and help minimize disparities in breast cancer detection and survival.

Mobile mammography users were less likely than users of fixed sites to understand their breast cancer risk or to perceive it accurately. Women with higher perceived breast cancer risk have been found to be more likely to obtain mammograms or adhere to screening mammogram guidelines (29,30). Women who underestimate their breast cancer risk, compared with women who overestimate or correctly estimate their breast cancer risk, tend to be from racial/ethnic minority backgrounds, have less income, and be less educated (18,29,30). Efforts to increase mammographic screening among underserved women could be enhanced by providing education on breast cancer risk. Such education is an area of research and public policy that should be addressed.

Our review has several limitations. First, we focused exclusively on published scientific literature. Other studies of mobile mammography may have been conducted but not published in scientific journals. The inclusion of scientific literature only may have led to publication bias, because studies with negative or null outcomes are less likely to be published. However, our use of studies in scientific journals only helped to ensure that the research examined was of reasonable quality. Second, most of our included studies were derived from programs conducted in the southern United States, which has a history of racial/ethnic health disparities. This factor could have skewed our results — particularly our finding that African American women are the underserved group most likely to use mobile mammography. Nevertheless, the ability of mobile mammography to reach underserved groups remains a key finding. Third, we found high rates of not having health insurance among mobile mammography users; it is not known whether such high rates will continue to prevail at mobile clinics. Although our included studies were published during implementation of the Affordable Care Act, some of the studies were conducted before the insurance mandate. However, given the interest among underserved women in using mobile mammography and the tenuous future of the Affordable Care Act, we believe mobile mammography will remain an important resource for women from underserved communities. Fourth, 7 of the 10 studies described mobile mammography clinics that were operated by university hospitals, which could have biased results and limited generalizability. Lastly, because no study conducted an intervention, we could not analyze pooled data to determine the effectiveness of the mobile mammography programs described.

Our findings have important implications for and highlight critical gaps in research on the use of mobile mammography among underserved populations. Mobile mammography programs can be used to resolve disparities in mammographic screening rates. Future efforts aimed at improving screening mammogram uptake should target women from low-income groups, women with low educational attainment, and women with no health insurance. Adding patient navigation to mobile mammography programs may help improve screening mammography completion and promote further evaluation of any resulting abnormal mammograms. Programs to promote screening should be delivered in a culturally congruent manner, and risk assessments should account for a woman’s racial/ethnic background. Efforts are needed to educate communities about the quality of mobile mammography, the importance of follow-up, and individual breast cancer risk. Future research should focus on understanding why many women do not return to mobile mammography clinics after their initial visit.

## References

[1] National Cancer Institute. Cancer stat facts: breast cancer. https://seer.cancer.gov/statfacts/html/breast.html. Accessed July 30, 2018.

[2] Sprague BL , Trentham-Dietz A , Gangnon RE , Ramchandani R , Hampton JM , Robert SA , Socioeconomic status and survival after an invasive breast cancer diagnosis. Cancer 2011;117(7):1542–51. 10.1002/cncr.25589 21425155 PMC3116955

[3] Morelli V . An introduction to primary care in underserved populations: definitions, scope, and challenges. Prim Care 2017;44(1):1–9. 10.1016/j.pop.2016.09.002 28164809

[4] Henry KA , Sherman R , Farber S , Cockburn M , Goldberg DW , Stroup AM . The joint effects of census tract poverty and geographic access on late-stage breast cancer diagnosis in 10 US States. Health Place 2013;21:110–21. 10.1016/j.healthplace.2013.01.007 23454732

[5] DeSantis CE , Ma J , Goding Sauer A , Newman LA , Jemal A . Breast cancer statistics, 2017, racial disparity in mortality by state. CA Cancer J Clin 2017;67(6):439–48. 10.3322/caac.21412 28972651

[6] Berg WA , Gutierrez L , NessAiver MS , Carter WB , Bhargavan M , Lewis RS , Diagnostic accuracy of mammography, clinical examination, US, and MR imaging in preoperative assessment of breast cancer. Radiology 2004;233(3):830–49. 10.1148/radiol.2333031484 15486214

[7] Ahmed AT , Welch BT , Brinjikji W , Farah WH , Henrichsen TL , Murad MH , Racial disparities in screening mammography in the United States: a systematic review and meta-analysis. J Am Coll Radiol 2017;14(2):157–165.e9. 10.1016/j.jacr.2016.07.034 27993485

[8] Calo WA , Vernon SW , Lairson DR , Linder SH . Area-level socioeconomic inequalities in the use of mammography screening: a multilevel analysis of the health of Houston survey. Womens Health Issues 2016;26(2):201–7. 10.1016/j.whi.2015.11.002 26809487 PMC4761271

[9] Stoll CR , Roberts S , Cheng MR , Crayton EV , Jackson S , Politi MC . Barriers to mammography among inadequately screened women. Health Educ Behav 2015;42(1):8–15. 10.1177/1090198114529589 24722216

[10] Corrarino JE . Barriers to mammography use for black women. J Nurse Pract 2015;11(8):790–6. 10.1016/j.nurpra.2015.05.016

[11] Liberati A , Altman DG , Tetzlaff J , Mulrow C , Gøtzsche PC , Ioannidis JP , The PRISMA statement for reporting systematic reviews and meta-analyses of studies that evaluate health care interventions: explanation and elaboration. PLoS Med 2009;6(7):e1000100. 10.1371/journal.pmed.1000100 19621070 PMC2707010

[12] Chen YR , Chang-Halpenny C , Kumarasamy NA , Venegas A , Braddock Iii CH . Perspectives of mobile versus fixed mammography in Santa Clara County, California: a focus group study. Cureus 2016;8(2):e494. 27014528 10.7759/cureus.494 PMC4792640

[13] Fayanju OM , Kraenzle S , Drake BF , Oka M , Goodman MS . Perceived barriers to mammography among underserved women in a Breast Health Center Outreach Program. Am J Surg 2014;208(3):425–34. 10.1016/j.amjsurg.2014.03.005 24908357 PMC4135000

[14] Stanley E , Lewis MC , Irshad A , Ackerman S , Collins H , Pavic D , Effectiveness of a mobile mammography program. AJR Am J Roentgenol 2017;209(6):1426–9. 10.2214/AJR.16.17670 28871806

[15] Vyas A , Madhavan S , Kelly K , Metzger A , Schreiman J , Remick S . Do Appalachian women attending a mobile mammography program differ from those visiting a stationary mammography facility? J Community Health 2013;38(4):698–706. 10.1007/s10900-013-9667-z 23504266 PMC4893946

[16] Brooks SE , Hembree TM , Shelton BJ , Beache SC , Aschbacher G , Schervish PH , Mobile mammography in underserved populations: analysis of outcomes of 3,923 women. J Community Health 2013;38(5):900–6. 10.1007/s10900-013-9696-7 23674194 PMC3765844

[17] Drake BF , Abadin SS , Lyons S , Chang SH , Steward LT , Kraenzle S , Mammograms on-the-go-predictors of repeat visits to mobile mammography vans in St Louis, Missouri, USA: a case-control study. BMJ Open 2015;5(3):e006960. 10.1136/bmjopen-2014-006960 25795693 PMC4368932

[18] LeMasters T , Madhavan S , Atkins E , Vyas A , Remick S , Vona-Davis L . “Don’t know” and accuracy of breast cancer risk perceptions among Appalachian women attending a mobile mammography program: implications for educational interventions and patient empowerment. J Cancer Educ 2014;29(4):669–79. 10.1007/s13187-014-0621-2 24563177 PMC4896074

[19] Mizuguchi S , Barkley L , Rai S , Pan J , Roland L , Crawford S , Mobile mammography, race, and insurance: use trends over a decade at a comprehensive urban cancer center. J Oncol Pract 2015;11(1):e75–80. 10.1200/JOP.2014.001477 25371543

[20] Roen EL , Roubidoux MA , Joe AI , Russell TR , Soliman AS . Adherence to screening mammography among American Indian women of the Northern Plains. Breast Cancer Res Treat 2013;139(3):897–905. 10.1007/s10549-013-2580-4 23749344 PMC3760373

[21] Vyas A , Madhavan S , LeMasters T , Atkins E , Gainor S , Kennedy S , Factors influencing adherence to mammography screening guidelines in Appalachian women participating in a mobile mammography program. J Community Health 2012;37(3):632–46. 10.1007/s10900-011-9494-z 22033614

[22] American Cancer Society. Breast cancer facts and figures 2017–2018. Atlanta (GA): American Cancer Society, Inc; 2017. https://www.cancer.org/content/dam/cancer-org/research/cancer-facts-and-statistics/breast-cancer-facts-and-figures/breast-cancer-facts-and-figures-2017-2018.pdf. Accessed August 6, 2018.

[23] US Department of Health and Human Services, Office of Disease Prevention and Health Promotion. Healthy people 2020: objectives. https://www.healthypeople.gov/2020/topics-objectives/topic/cancer/objectives. Accessed August 6, 2018.

[24] Narayan A , Fischer A , Zhang Z , Woods R , Morris E , Harvey S . Nationwide cross-sectional adherence to mammography screening guidelines: National Behavioral Risk Factor Surveillance System Survey results. Breast Cancer Res Treat 2017;164(3):719–25. 10.1007/s10549-017-4286-5 28508184

[25] Oeffinger KC , Fontham ETH , Etzioni R , Herzig A , Michaelson JS , Shih YC , . Breast cancer screening for women at average risk: 2015 guideline update from the American Cancer Society. JAMA 2015;314(15):1599–614. 10.1001/jama.2015.12783 26501536 PMC4831582

[26] Kidd AD , Colbert AM , Jatoi I . Mammography: review of the controversy, health disparities, and impact on young African American women. Clin J Oncol Nurs 2015;19(3):E52–8. 10.1188/15.CJON.E52-E58 26000591

[27] Moyce SC , Schenker M . Migrant workers and their occupational health and safety. Annu Rev Public Health 2018;39(1):351–65. 10.1146/annurev-publhealth-040617-013714 29400993

[28] Shah SJ , Cronin P , Hong CS , Hwang AS , Ashburner JM , Bearnot BI , Targeted reminder phone calls to patients at high risk of no-show for primary care appointment: a randomized trial. J Gen Intern Med 2016;31(12):1460–6. 10.1007/s11606-016-3813-0 27503436 PMC5130951

[29] Katapodi MC , Lee KA , Facione NC , Dodd MJ . Predictors of perceived breast cancer risk and the relation between perceived risk and breast cancer screening: a meta-analytic review. Prev Med 2004;38(4):388–402. 10.1016/j.ypmed.2003.11.012 15020172

[30] Elewonibi BR , Thierry AD , Miranda PY . Examining mammography use by breast cancer risk, race, nativity, and socioeconomic status. J Immigr Minor Health 2018;20(1):59–65. 10.1007/s10903-016-0502-3 27662888

